# Application of freeze-dried *Yarrowia lipolytica* biomass in the synthesis of lipophilic antioxidants

**DOI:** 10.1007/s10529-020-03033-6

**Published:** 2020-10-26

**Authors:** Bartłomiej Zieniuk, Małgorzata Wołoszynowska, Ewa Białecka-Florjańczyk, Agata Fabiszewska

**Affiliations:** 1 Department of Chemistry, Institute of Food Sciences, Warsaw University of Life Sciences, 159c Nowoursynowska St., 02-776 Warsaw, Poland; 2 Analytical Department, Łukasiewicz Research Network - Institute of Industrial Organic Chemistry, 6 Annopol St., 03-236 Warsaw, Poland

**Keywords:** *Candida antarctica* lipase B, Enzymatic synthesis, Lipophilic antioxidants, Phenolic acids esterification, *Yarrowia lipolytica*

## Abstract

**Objective:**

The aim of the study was to evaluate the possibility of using *Y. lipolytica* biomass as a whole-cell catalyst in the synthesis of lipophilic antioxidants, with the example of esterification of five phenolic acids with 1-butanol.

**Results:**

Freeze-dried *Y. lipolytica* biomass was successfully applied as a biocatalyst in the synthesis of esters of phenylpropanoic acid derivatives with 75–98% conversion. However, in the case of phenylacetic acid derivatives, results below 10% were obtained. The biological activity of phenolic acid esters was strongly associated with their chemical structures. Butyl 3-(4-hydroxyphenyl)propanoate showed an IC_50_ value of 19 mg/ml (95 mM) and TEAC value of 0.427. Among the compounds tested, butyl esters of 3-(4-hydroxyphenyl)propanoic and 4-hydroxyphenylacetic acids exhibited the highest antifungal activity.

**Conclusions:**

Lipophilization of phenolic acids achieved by enzymatic esterification creates prospects for using these compounds as food additives with antioxidant properties in lipid-rich food matrices.

## Introduction

Free radicals are atoms, ions, or molecules with one or more unpaired electrons capable of independent existence. High levels of instability and reactivity increase their ability to cause damage in various molecular structures, and the overproduction of reactive oxygen species (ROS) leads to oxidative stress, a deleterious process contributing to the development of many civilization diseases (Valko et al. 2007).

**Fig. 1:**
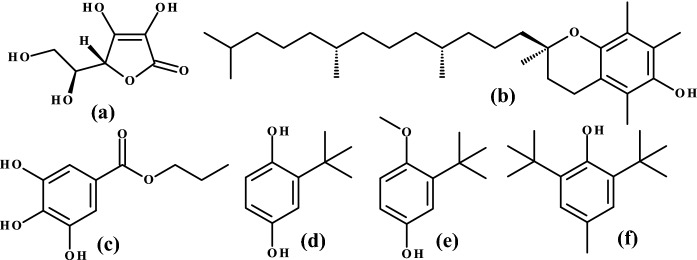
Chemical structures of selected antioxidants used as food additives: **a** l-ascorbic acid, **b** (+)-α-tocopherol; **c** propyl gallate; **d** TBHQ; **e** BHA; **f** BHT

The term "antioxidant" refers to compounds capable of neutralizing free radicals. Antioxidants are responsible for stabilizing color and prolonging the durability of food products and, therefore, are important additives in food technology. These compounds play a major role in preventing oxidation processes in frozen and refrigerated food, as well as lowering the oxidative effect of sunlight and oxygen in food products (Carocho et al. 2014). Antioxidant food additives permitted for use within the European Union are ascorbic acid, and its salts, esters, and tocopherols, as well as propyl gallate, *tert*-butylhydroquinone (TBHQ), butylated hydroxyanisole (BHA), and butylated hydroxytoluene (BHT) (Silva and Lidon 2016) (Fig. 1).

Oils and fats, especially those with high unsaturated fatty acid contents, are food ingredients susceptible to oxidation processes (Yun and Surh 2012). Therefore, lipophilic antioxidants are desirable additives in food technology. In industrial practice, synthetic compounds, like TBHQ, BHT, and BHA, are most often used, despite the fact that they arouse controversy among consumers. As a consequence, bioactive substances from natural sources or identical additives with similar properties to synthetic ones are being sought (Abdelazim et al. 2013). Given this, lipophilic antioxidants synthesized by biotechnological methods are gaining popularity. This method usually involves enzymatic esterification of suitable compounds with high antioxidant activity and a lipophilic moiety (fatty acid or long-chain alcohol), resulting in lipid-soluble esters that inhibit oxidation of food components, as in the case of fatty acid esters of ascorbic acid (Viklund et al. 2003) or alkyl esters of phenolic acids (Białecka-Florjańczyk et al. 2018). Moreover, exploration of new food additives is an issue focusing on scientific attention and industrial interest. The most required are molecules synthesized in a process that reduces or eliminates the use or generation of substances that are hazardous to humans and the environment. Enzyme-catalyzed synthesis of biologically active compounds, compared to traditional chemical catalysis, has many advantages, such as less environmental pollution, lower energy consumption, fewer by-products, and milder reaction conditions (Anastas and Zimmerman 2018).

*Yarrowia lipolytica* yeast is capable of producing many metabolites of application importance, especially hydrolytic enzymes such as lipases, proteases, phosphatases, or RNases (Zieniuk and Fabiszewska 2019). In addition, it produces many other valuable compounds with high biological activity. Some compounds of interest exhibited antimicrobial and/or antioxidant roles. For example, Morgunov et al. (2018, 2019) presented the antioxidant effect of isocitric acid. This metabolite produced by *Y. lipolytica* was a more efficient antioxidant than ascorbic acid in studies with *Paramecium caudatum* stressed by hydrogen peroxide and salts of heavy metals, such as Cu, Pb, Zn, and Cd (Morgunov et al. 2018). Furthermore, isocitric acid mitigated the neurotoxic effect of lead and molybdenum salts, which was noticed after the observation of beneficial learning effect in rats intoxicated with the aforementioned heavy metal salts (Morgunove et al. 2019). Moreover, antimicrobial and nematodic effects of succinic acid produced in a two-step process in the culture of *Y. lipolytica* were evaluated by Kamzolova et al. (2014). Succinic acid inhibited the growth of *Staphylococcus aureus*, *Erwinia carotovora*, and *Penicillium casei*, as well as inactivating 72% of *Ditylenchus destructor*, a potato rot nematode (Kamzolova et al. 2014).

The aim of this study was to evaluate the possibility of using the whole-cell catalyst of *Y. lipolytica* biomass, compared with commercially available *Candida antarctica* lipase B in the esterification of lipophilic derivatives of some phenolic acids and 1-butanol.

Phenolic compounds are well-known free radical scavengers, found mainly in plant extracts (Białecka-Florjańczyk et al. 2018). Regarding usefulness in food technology, their main disadvantage is low solubility in lipid-rich matrices which can be overcome by enzyme-catalyzed esterification (Figueroa-Espinoza and Villeneuve 2005). Additionally, the derivatization of a substrate could grant new antioxidant and antimicrobial activities. Organic molecules, synthesized in mild conditions under sustainable chemical procedures, that preserve food and enlarge the availability of high-quality food, are undoubtedly desired products. Finally, compounds characterized by antimicrobial and antioxidant activity may enable the acquisition of safe food, free from food spoilage microorganisms, and free radicals that cause adverse changes in food (Shi et al. 2018, 2019). Importantly, enzymatic synthesis methods open new ways of producing valuable products under mild conditions in contrast to traditional chemical methods (Pradima et al. 2017).

## Material and methods

### Microorganism

In the study following yeast and bacterial strains were used: *Y. lipolytica* KKP 379, *Candida cylindracea* DSM-2031, *Rhodotorula mucilaginosa, Saccharomyces cerevisiae*, *Escherichia coli* PCM 2057, *Pseudomonas aeruginosa* PCM 2058, *Staphylococcus aureus* PCM 2054, and *Bacillus subtilis* PCM 486. *Y. lipolytica* KKP 379 was purchased from the Collection of Industrial Microorganisms of Institute of Agricultural and Food Biotechnology in Warsaw. Bacterial strains were purchased from Polish Collection of Microorganisms (PCM) of Institute of Immunology and Experimental Therapy Polish Academy of Sciences (Wrocław, Poland). *C. cylindracea* was purchased from Leibniz Institute DSMZ – German Collection of Microorganisms and Cell Cultures. *R. mucilaginosa* and *S. cerevisiae* were isolated and identified in Department of Chemistry (WULS, Poland). Prior to testing yeasts and bacterial strains were stored at − 20 °C using Protect Microorganism Preservation System (Technical Service Consultants Ltd, Great Britain).

### Materials

*Candida antarctica* Lipase B (CALB, product number: L4777) was purchased from Sigma-Aldrich (Poznań, Poland). Chemicals were purchased from Sigma-Aldrich and Avantor Performance Materials (Gliwice, Poland). Culture media components were purchased from BTL Sp. z o. o. (Łódź, Poland), and extra virgin olive oil (Aceites Borges Pont, Tarrega, Spain), which was used as a carbon source in the cultivation of *Y. lipolytica* was purchased from the local supermarket in Warsaw (Poland).

### Obtaining *Y. lipolytica* biomass as a whole-cell biocatalyst

Freeze-dried whole-cell biocatalyst was obtained as described previously (Stolarzewicz et al. 2017). Briefly, yeasts were cultivated in BIOFLO 3000 bioreactor (New Brunswick Scientific Edison, NJ, USA) in 4 L of YPO medium (2% peptone, 1% yeast extract and 2% olive oil) at 28 °C, 300 rpm of agitator speed and compressed air aeration. Bioreactor culture was ended upon reaching relatively high dry mass content and high activity of intracellular lipolytic enzymes (Stolarzewicz et al. 2017). Yeast biomass was then separated by centrifugation (10 °C, 8 000 rpm, 10 min), washed twice by 0.9% sodium chloride solution and freeze-dried for 24 h using Christ Gamma 1–16 laboratory freeze dryer (Germany). The process was conducted at a shelf temperature of 10 °C and the pressure inside the chamber was kept at 63 Pa. Freeze-dried yeast biomass was characterized by the following determinations: dry mass and moisture measured by Radwag MAC 50/NH (Poland) moisture analyzer at 105 °C, water activity determined using AquaLab CX-2 (USA) water activity meter with dew point sensor at 25 °C, protein content determined by Kjeldahl method and intracellular lipase activity measured by a spectrophotometric method based on the hydrolysis of *p*-nitrophenyl laurate (Kapturowska et al. 2012). These values are summarized in Table 1.

**Table 1:** Characteristics of freeze-dried biocatalyst from *Y. lipolytica* KKP 379

Dry matter (%)	Moisture (%)	Water activity	Protein content (mg/g)	Lipase activity (U/g)	Specific activity (U/mg)
98.0 ± 0.2	2.0 ± 0.2	0.090 ± 0.006	530 ± 1	49.12 ± 3.26	0.093

### Biotransformation reaction

**Fig. 2:**
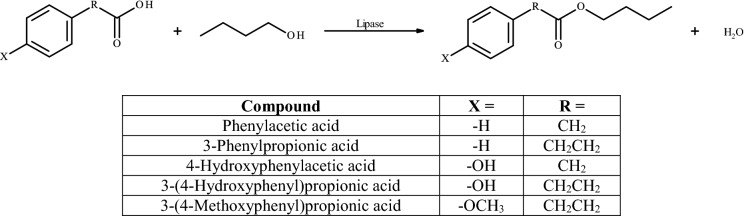
Reaction scheme of enzymatic esterification of phenolic acids with 1-butanol. X = –H, –OH or –OCH_3_; R = CH_2_ or CH_2_CH_2_

Following phenolic acids and their analogs were used: phenylacetic acid, 3-phenylpropionic acid, 4-hydroxyphenylacetic acid, 3-(4-hydroxyphenyl)propionic acid, and 3-(4-methoxyphenyl)propionic acid. 1-Butanol was chosen as a second reaction substrate. Reaction scheme of enzymatic esterification of phenolic acids with 1-butanol was presented in Fig. 2. Substrates were applied in molar ratio 1:2 (acid: alcohol) and the amount of acid was 0.0025 mol. *C. antarctica lipase* B (5% w/w of substrates mass) and freeze-dried *Y. lipolytica* biomass (2 g) were used as biocatalysts. Reactions were carried out in Erlenmeyer glass flasks (100 ml) in 10 ml of a mixture of isooctane and methyl-*tert*-butyl ether (7:3, (v/v)) using an orbital shaker (200 rpm) at 28 °C. Liquid samples were withdrawn after 1, 2 and 24 h from the reaction mixtures and analyzed by gas chromatography (GC).

### Gas chromatography

Samples were derivatized with pyridine and BSTFA (*N*,*O*-bis(trimethylsilyl)trifluoroacetamide) + 1% TMCS (trimethylchlorosilane) added in equal volumes and heated for 30 min at 70 °C. Then samples were analyzed by gas chromatography equipped with a flame ionization detector (GC-FID). Analysis was carried out using Agilent Technologies 7820A with HP-5 column (0.25 mm, 30 m, 0.25 µm). Nitrogen was used as carrier gas at a flow rate of 1.5 ml/min. The temperature program was as follow: 70 °C for 3 min, 70 °C to 150 °C (3 °C/min), 150 °C to 300 °C (40 °C/min), and 300 °C for 10 min. Injector temperature: 250 °C and detector temperature: 290 °C, injection volume: 1 µl. Percentage conversion was calculated based on the area under peaks of acid and ester.

### Column chromatography

Esters were purified using column chromatography (chloroform was used as eluent in the case of butyl phenylacetate and butyl 3-phenylpropanoate; and heptane: ethyl acetate (3:1 v/v) mixture was applied for the purification of the remaining esters). The ^1^H NMR spectra of esters were measured using Bruker AVANCE 300 MHz and CDCl_3_ as a solvent.

Butyl phenylacetate: ^1^H NMR (300 MHz, CDCl_3_): δ 0.94 (3H, t, *J* = 7.3 Hz), 1.37 (2H, m), 1.63 (2H, m), 3.64 (2H, s), 4.12 (2H, t, *J* = 6.7 Hz), 7.15–7.30 (5H, m).

Butyl 3-phenylpropanoate: ^1^H NMR (300 MHz, CDCl_3_): δ 0.94 (3H, t, *J* = 7.3 Hz), 1.37 (2H, m), 1.63 (2H, m), 2.65 (2H, t, *J* = 7.8 Hz), 2.98 (2H, t, *J* = 7.8 Hz), 4.12 (2H, t, *J* = 6.7 Hz), 7.16–7.38 (5H, m).

Butyl 4-hydroxyphenylacetate: ^1^H NMR (300 MHz, CDCl_3_): δ 0.94 (3H, t, *J* = 7.4 Hz), 1.37 (2H, m), 1.63 (2H, m), 3.64 (2H, s), 4.12 (2H, t, *J* = 6.7 Hz), 5.60 (1H, s), 6.70–6.81 (2H, m), 7.08–7.19 (2H, m).

Butyl 3-(4-hydroxyphenyl)propanoate: ^1^H NMR (300 MHz, CDCl_3_): δ 0.94 (3H, t, *J* = 7.3 Hz), 1.37 (2H, m), 1.63 (2H, m), 2.65 (2H, t, *J* = 7.8 Hz), 2.98 (2H, t, *J* = 7.8 Hz), 4.09 (2H, t, *J* = 6.6 Hz), 4.99 (1H, s), 6.71–6.82 (2H, m), 7.03–7.14 (2H, m).

Butyl 3-(4-methoxyphenyl)propanoate: ^1^H NMR (300 MHz, CDCl_3_): δ 0.94 (3H, t, *J* = 7.3 Hz), 1.37 (2H, m), 1.63 (2H, m), 2.65 (2H, t, *J* = 7.8 Hz), 2.98 (2H, t, *J* = 7.8 Hz), 3.81 (3H, s), 4.09 (2H, t, *J* = 6.7 Hz), 6.74–6.85 (2H, m), 7.09–7.20 (2H, m).

### Evaluation of antimicrobial properties

Antimicrobial activity of synthesized esters was evaluated using the disk diffusion method with Mueller–Hinton agar (BD, Germany) or Sabouraud dextrose agar (BTL Sp. z o. o., Łódź, Poland) plates. Blank 6 mm discs (Oxoid, UK) were soaked with 10 µL of the compound. Bacterial or yeasts suspensions in 0.9% NaCl solution at a density of 0.5 McFarland were spread over the surface of the agar plates. Then discs were placed on the agar and plates were incubated for 16–18 h at 37 °C (bacteria) or 48 h at 28 °C (in case of yeasts). After incubation, inhibition zone diameters were measured.

### Evaluation of antioxidant activity

The antioxidant activity of esters and their precursors was carried out using DPPH (2,2-diphenyl-1-picrylhydrazyl) method following Zanetti et al. (2017). The percentage reduction of the DPPH radical was determined after 60 min. Moreover, the IC_50_ parameter (concentration required for 50% reduction of the DPPH radical) was determined. Additionally, CUPRAC (cupric reducing antioxidant capacity) method was carried out following Apak et al. (2008) with neocuproine as a reagent. Trolox equivalent antioxidant capacities (TEAC) were determined for esters and their precursors.

### Prediction of the partition coefficient and solubility in water of obtained esters and their precursors

To calculate physicochemical properties of the compounds Osiris DataWarrior was used (DataWarrior V5.0.0, Idorsia Pharmaceuticals Ltd., Allschwil, Switzerland). Following parameters were calculated: partition coefficient (lipophilicity, expressed as logP) and solubility in water (expressed as logS, where S it is water solubility in mol/l, pH 7.5 and 25 °C).

### Statistical analysis

Analyses were performed using Statistica 13.3 software (TIBCO Software Inc., Palo Alto, CA, USA). One-way analysis of variance (ANOVA) was used to measure the significance of the main effects. Tukey’s test was used to determine significant differences among means (p < 0.05).

## Results and discussion

### Comparison of biocatalysts in phenolic acids esterification

**Fig. 3:**
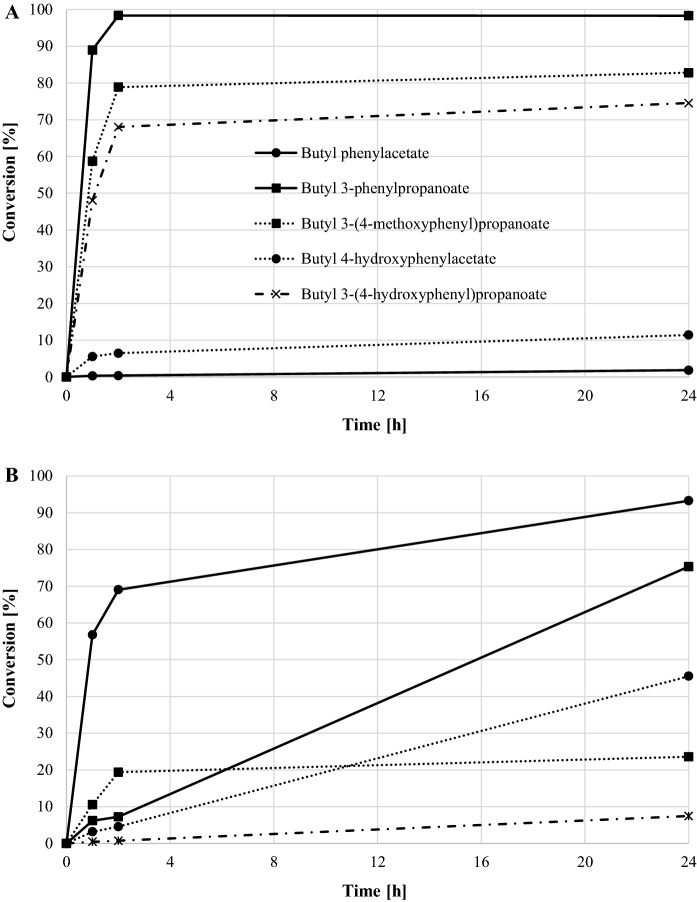
Percentage conversion of phenolic acids to butyl esters with following catalyst: **a** freeze-dried *Y. lipolytica* biomass, **b** *C. antarctica* lipase B (CALB)

Freeze-dried biomass of *Y. lipolytica* was used in the esterification of five different phenolic acids with 1-butanol. *C. antarctica* lipase B was used as a comparison. Conversion of phenolic acids to butyl esters is presented in Fig. 3. Biomass-catalyzed esterification was most effective in reactions with 3-phenylpropionic acid and its derivatives containing a hydroxy or methoxy group in the aromatic ring. The highest conversion rate was obtained after 2 h of reaction in the synthesis of butyl phenylpropanoate (98%). After 24 h, butyl 3-(4-hydroxyphenyl)propanoate and butyl 3-(4-methoxyphenyl)propanoate conversions were 75 and 83%, respectively. Biomass-catalyzed reactions, in which the substrate was phenylacetic or 4-hydroxyphenylacetic acid, were ineffective.

Using the CALB as a catalyst, an opposite trend in reactivity of tested phenolic acids was observed. The highest conversion (93% after 24 h) was achieved in a reaction with phenylacetic acid (completely unreactive in the presence of freeze-dried *Y. lipolytica* biomass), and in the case of 4-hydroxyphenylacetic acid, maximum conversion was 46% (about 10% with freeze-dried *Y. lipolytica* biomass). 3-phenylpropionic acid was less reactive than in the previous case, and 75% conversion was achieved after 24 h. For the other two substituted 3-phenylpropionic acids, the esterification yield did not exceed 25%.

Differences in conversion between the two biocatalysts resulted from their substrate specificity. Lipases are enzymes belonging to the class of hydrolases (EC 3.1.1.3) and are responsible for the hydrolysis of ester bonds in triacylglycerol molecules. In 1985, Zaks and Klibanov described that lipases can act in nearly anhydrous environments and catalyze reactions inverse to hydrolysis, such as esterification, transesterification, aminolysis, etc. (1985). Pleiss et al. (1998) described binding sites of eight serine esterases and lipases. Further, specificity of the substrate is related to the shape of the acyl binding site. Esterases and lipases differ in shape, depth, and physico-chemical properties of the substrate-binding pocket. Esterases have a small acyl binding pocket that optimally matches the acyl moiety of their substrates (Pleiss et al. 1998).

*C. antarctica* lipase B, as a representative of lipases, has a substrate binding pocket in the shape of an elliptical, steep funnel and shows higher activity for short- and medium-chain fatty acids than long-chain fatty acids. Reducing the size of the binding site can lead to steric conflicts with the substrate, but on the other hand, its larger size leads to suboptimal binding of the substrate due to the presence of free space (Pleiss et al. 1998). Juhl et al. (2010) suggested that CALB catalyzes reactions with a broad spectrum of alcohols. However, it is specific for acids, where reactions with straight-chain fatty acids proceed with high yield, and branched fatty acids significantly reduce reaction yield.

Fatty acids are linear and flexible molecules, whereas compounds like sugar acids (Otto et al. 1998) or phenolic compounds with a carboxyl group and other substituents (Otto et al. 2000) represent bulky acyl donors. Otto et al. (2000) examined substrate specificity of CALB in the synthesis of arylaliphatic glycolipids. Molecular modeling was used, and based on this computer-aided investigation, researchers claimed that at least one methylene bridge between the carboxylic group and aromatic ring is necessary for the reactions to proceed. Assumedly, this has to do with the molecule's flexibility, and thus, the possibility of it adapting to the hydrophobic cavity (Santos et al. 2019). Moreover, unsaturated phenolic acids (cinnamic, *p*-coumaric, and caffeic), as well as α-substituted phenolic acids are not suitable substrates for CALB esterification (Otto et al. 2000).

In the current study, esterification reactions of phenylacetic or 3-phenylpropionic acid (and their analogs substituted in the *para* position) with linear alcohol were studied, and similar observations to Otto et al. (2000) were made both for CALB and *Y. lipolytica* whole-cell biocatalyst. Reduced conversions were obtained in reactions with substrates containing hydroxy or methoxy substituents in the aromatic ring, and when using unsubstituted analogs higher conversions were achieved.

Substrates containing at least two carbon atoms between the aromatic ring and the carboxyl group were preferred by enzymes present in *Y. lipolytica* biomass (a definite difference in the conversion of 3-phenylpropionic acid compared to phenylacetic acid). Thus, describing substrate specificity of *Y. lipolytica* intracellular enzymes using whole-cell biocatalyst is not easy. The authors considered the involvement, in the catalytic process, of some intracellular lipases of *Y. lipolytica* found in the cytosol or associated with cell wall structures, but there is still little knowledge about them and their substrate specificity (Fickers et al. 2011). According to Fickers et al. (2005), Lip7p and Lip8p are lipases associated to the cell wall, but they are easily released by washing cells with phosphate buffer. *LIP1* and *LIP3* genes have been also described, with products that are probably intracellular or membrane-bound enzymes belonging to the carboxylesterase family (Pignede et al. 2000). Dulermo et al. (2013) characterized two intracellular lipases of *Y. lipolytica* encoded by *TGL3* and *TGL4* genes involved in triacylglycerols degradation in lipid bodies. YlTgl4 is a typical serine hydrolase, but YlTgl3 is not and may act as a regulator of YlTgl4 (Dulermo et al. 2013).

### Lipohilization of phenolic compounds by esterification

Esterification yielded compounds more soluble in organic environments, which was confirmed by calculations of the partition coefficient and logarithm of solubility, given in Table 2. Compounds with logP between 1 and 3 are characterized as being lipophilic enough to cross the blood–brain barrier, which predetermines chemicals as promising antioxidant agents in the treatment of neurodegenerative diseases. All synthesized esters were characterized by beneficial logP and logS parameters regarding their solubility in fat-rich matrices.

**Table 2:** Antioxidant activity of phenolic acids and their butyl esters measured in DPPH and CUPRAC methods, and predicted partition coefficient (logP) and solubility (logS) of compounds

Compound	DPPH			CUPRAC	logP	logS
	AA (%) ± SD	IC_50_ (mg/ml)	IC_50_ (mM)	TEAC ± SD		
Phenylacetic acid	2.08 ± 0.84^e^	NA	NA	0.023 ± 0.003^e^	1.14	− 1.60
Butyl phenylacetate	2.25 ± 0.11^e^	NA	NA	0.021 ± 0.002^e^	2.89	− 2.56
3-Phenylpropionic acid	2.81 ± 0.34^e^	NA	NA	0.019 ± 0.001^e^	1.60	− 1.86
Butyl 3-phenylpropanoate	5.57 ± 0.06^d^	NA	NA	0.020 ± 0.003^e^	3.34	− 2.83
4-Hydroxyphenylacetic acid	41.73 ± 0.45^b^	26	170	0.504 ± 0.011^b^	0.80	− 1.30
Butyl 4-hydroxyphenylacetate	25.31 ± 0.67^c^	43	205	0.163 ± 0.007^d^	2.54	− 2.27
3-(4-Hydroxyphenyl)propionic acid	51.86 ± 2.02^a^	18	108	0.579 ± 0.017^a^	1.25	− 1.57
Butyl 3-(4-hydroxyphenyl)propanoate	50.62 ± 0.67^a^	19	85	0.427 ± 0.016^c^	2.99	− 2.54
3-(4-Methoxyphenyl)propionic acid	1.86 ± 0.17^e^	NA	NA	0.031 ± 0.005^e^	1.53	− 1.88
Butyl 3-(4-methoxyphenyl)propanoate	2.14 ± 0.11^e^	NA	NA	0.030 ± 0.005^e^	3.27	− 2.85

Reis et al. (2010) compared the antioxidant activity of protocatechuic acid and its three esters: methyl, ethyl, and propyl. Esterification of phenolic acid with linear alcohol increased the solubility of ester derivatives of protocatechuic acid in apolar media, which also seemed to affect stabilization of the radicals formed in the oxidation processes, which was studied by DSC measurements with linoleic acid (Reis et al. 2010). Notably, lipophilization can only be carried out by esterification of the carboxyl group and is not applicable to phenolic function, which determines a compound's antioxidant properties (Velika and Kron 2012).

### Evaluation of antioxidant activities of synthesized esters

Compounds obtained in esterification reactions and the substrates used for their synthesis were tested for antioxidant activity in DPPH and CUPRAC methods. Compounds containing a hydroxy group in the phenyl ring showed the highest antioxidant activity. The most effective DPPH radical scavengers were 3-(4-hydroxyphenyl)propionic acid and its butyl ester, with antioxidant activities around 50%. Also, 4-hydroxyphenylacetic acid was an effective compound, with about 42% activity, and its butyl ester activity was 1.5-fold lower. For other compounds, antioxidant activity did not exceed 10%. IC_50_ values were also determined for the four most active chemical compounds. Concentrations were expressed in two different units, mg/ml and mM, which takes the molecular weight of the compound into account. The esterification of 3-(4-hydroxyphenyl)propionic acid did not significantly affect the antioxidant activity, unlike 4-hydroxyphenylacetic acid and its ester, where the ester was much less active. The obtained results of antioxidant activity with the DPPH radical were comparable to the CUPRAC method. Compounds were characterized by the highest TEAC values in the following order: 3-(4-hydroxyphenyl)propionic acid > 4-hydroxyphenylacetic acid > butyl 3-(4-hydroxyphenyl)propanoate >  > butyl 4-hydroxyphenylacetate >  >  >  > the remaining compounds. The following TEAC values were obtained for the three most active compounds: 0.579, 0.504, and 0.427, respectively.

The activity of phenolic acids in free radical scavenging is associated with the number and position of hydroxy groups bonded to the aromatic ring. Thus, the more hydroxy groups a compound has, the greater its antioxidant activity. Furthermore, compounds with a hydroxy group in *ortho* or *para* positions to the carboxylic group showed better antioxidant properties, due to their hydrogen donating capacity against radicals (Velika and Kron 2012). Derivatives of cinnamic acid (i.e. *p*-coumaric, caffeic and ferulic acids) were more potent than hydroxybenzoic acid and its derivatives, due to the better stabilization of radical structures in view of the presence of a double bond conjugated with the aromatic ring (Białecka-Florjańczyk et al. 2018).

Roleira et al. (2010) acknowledged data obtained in the current study (Table 2) and showed that esters of phenolic acids had lower activity in the test with the DPPH radical or other methods where polar solvents were used (methanol or ethanol in the DPPH method, aqueous solution in the ABTS method and ethanol in the CUPRAC method used in the current study). On the other hand, hexyl esters of caffeic and ferulic acids (or their saturated analogs) showed higher activity in lipoperoxidation assay, where an organic environment occurred, compared to non-esterified acids (Roleira et al. 2010).

### Evaluation of antimicrobial activities of synthesized esters

Antimicrobial activity of the obtained esters was also determined due to the rising problem of foodborne pathogens and their drug resistance. Dual-functioning compounds (antioxidant and antimicrobial) may become useful as multi-task food additives in the near future. Esters without any group or with a methoxy group in phenyl ring did not possess antibacterial activity, and inhibition zone diameters were 8 mm maximum (Table 3). Only esters with hydroxy groups inhibited bacterial growth. Butyl 4-hydroxyphenylacetate, in the range of 12–14 mm and butyl 3-(4-hydroxyphenyl)propanoate inhibited *P. aeruginosa* PCM 2058 and *S. aureus* PCM 2054 growth (12 mm diameter). In the case of *E. coli* PCM 2057 and *B. subtilis* PCM 486, butyl 3-(4-hydroxyphenyl)propanoate was ineffective. A similar trend was observed for compounds that acted on yeast. Butyl 3-(4-methoxyphenyl)propanoate did not show any antimicrobial activity. Butyl esters of phenylacetic and 3-phenylpropionic acids weakly inhibited yeast growth. Yeasts were more sensitive than bacteria, and *C. cylindracea* DSM-2031 and *R. mucilaginosa* were the most sensitive tested microorganisms, where inhibition zone diameters for esters of 4-hydroxypheynlacetic and 3-(4-hydroxyphenyl)propionic acids exceeded 20 mm.

**Table 3:** Comparison of antimicrobial activity of the obtained esters against bacteria and yeasts

Compound	Microorganism							
	*E. coli* PCM 2057	*P. aeruginosa* PCM 2058	*S. aureus* PCM 2054	*B. subtilis* PCM 486	*Y. lipolytica* KKP 379	*C. cylindracea* DSM-2031	S. cerevisiae	R. mucilaginosa
	Inhibition zone [mm]							
Butyl phenylacetate	6*	8	6	8	8	10	6	10
Butyl 3-phenylpropanoate	6	8	6	8	8	12	6	6
Butyl 4-hydroxyphenylacetate	14	14	14	12	20	28	24	24
Butyl 3-(4-hydroxyphenyl)propanoate	6	12	12	8	14	26	18	20
Butyl 3-(4-methoxyphenyl)propanoate	6	6	6	6	6	6	6	6

*Growth inhibition zone of 6 mm means that compound did not inhibit the growth of the microorganism

Antioxidant and antimicrobial properties of phenolic acids and their derivatives (esters, amides, etc.) are a broadly studied issue, investigated most frequently for cinnamic acid and its phenolic derivatives. Shi et al. (2018, 2019) obtained six different esters of ferulic acid through lipase-catalyzed reactions. Of the compounds tested, hexyl ferulate proved to be the most active compound in inhibiting *E. coli* and *Listeria monocytogenes* growth. Researchers showed that the examined ester was able to lyse cells, disrupt cell membranes, and also, affect the protein expression system, causing changes in membrane protein conformations and contents (Shi et al. 2018, 2019). Similarly, Merkl et al. (2010) synthesized methyl, ethyl, propyl, and butyl esters of protocatechuic, gentisic, ferulic, caffeic, vanillic, and *p*-hydroxybenzoic acids, and then, antimicrobial activity was investigated. Butyl esters of phenolic acids were more active than other compounds, and an increase in the alkyl chain length increased antimicrobial activity. Among cinnamic acids, a similar tendency to exhibit greater antimicrobial activity against fungi and gram-positive bacteria compared to gram-negative bacteria was observed (Guzman 2014). According to the results of QSAR (quantitative structure–activity relationship) studies, derivatives of cinnamic acids with bulky aromatic groups or anilides were concluded to be more potent than other derivatives (Bisogno et al. 2007; Khatkar et al. 2017). Thus, the presence of electron-withdrawing substituents in compounds, which are responsible for higher antimicrobial activity, is important. Secondly, compounds with a double bond between the aromatic ring and carboxylic group are stronger antimicrobials than saturated compounds, due to their conformational and electronic characteristics.

Contrary to hydroxycinnamates, esters of phenylacetic, 3-phenylpropionic acids and their hydroxy derivatives are less studied. Cueva et al. (2010) examined antimicrobial activity of thirteen phenolic acids (benzoic, phenylacetic, and 3-phenylpropionic acids derivatives) towards selected bacteria and yeast. Unlike results in the current study, Cueva et al. (2010) indicated that both non-substituted benzoic, phenylacetic, and phenylpropionic acids were more potent antimicrobials to *E. coli* than phenolics with hydroxy and methoxy substituents. On the other hand, one comparable observation has been made in the current study—compounds with shorter side chain length (phenylacetic > phenylpropionic) have higher antimicrobial activity.

## Conclusion

In the current study, *Y. lipolytica* KKP 379 was confirmed to be capable of producing intracellular lipolytic enzymes that catalyzed esterification reactions in organic solvents. Furthermore, these enzymes have significant substrate specificity—freeze-dried *Y. lipolytica* biomass can be used as a biocatalyst in the synthesis of esters of selected phenolic acids. In particular, esters of phenylpropanoic acid derivatives were synthesized with 75–98% yield. However, in the case of phenylacetic acid, results below 10% were obtained.

The biological activity of phenolic acid esters is strongly associated with their chemical structure, i.e. the presence of substituents in the benzene ring. The hydroxy group is responsible for the antioxidant and antimicrobial activities, and among the obtained compounds, butyl 3-(4-hydroxyphenyl)propanoate showed a similar IC_50_ value (19 mg/ml; 95 mM) to its precursor—3-(4-hydroxyphenyl)propanoic acid (18 mg/ml; 108 mM) and TEAC values of 0.427 and 0.579, respectively. Considering its better solubility in lipid substances, this compound has prospects for use as a food additive with antioxidant properties. Moreover, butyl esters of 3-(4-hydroxyphenyl)propanoic and 4-hydroxyphenylacetic acids exhibited high antifungal activity, and butyl 4-hydroxyphenylacetate was more active against bacteria than butyl 3-(4-hydroxyphenyl)propanoate, as determined by the larger zones of growth inhibition.

Further research relating to the enzymatic synthesis of phenolic esters may allow the development of alternative methods for the production of food additives, which is economical and environmentally friendly.
